# HMGB1 promotes ERK-mediated mitochondrial Drp1 phosphorylation for chemoresistance through RAGE in colorectal cancer

**DOI:** 10.1038/s41419-018-1019-6

**Published:** 2018-09-26

**Authors:** Chih-Yang Huang, Shu-Fen Chiang, William Tzu-Liang Chen, Tao-Wei Ke, Tsung-Wei Chen, Ying-Shu You, Chen-Yu Lin, K. S. Clifford Chao, Chih-Yang Huang

**Affiliations:** 1 Translation Research Core, China Medical University Hospital, China Medical University, 40402 Taichung, Taiwan; 2 Department of Nutrition, HungKuang University, 43302 Taichung, Taiwan; 3 Cancer Center, China Medical University Hospital, China Medical University, 40402 Taichung, Taiwan; 4 Department of Colorectal Surgery, China Medical University Hospital, China Medical University, 40402 Taichung, Taiwan; 5 Department of Pathology, China Medical University Hospital, China Medical University, 40402 Taichung, Taiwan; 6 Graduate Institute of Basic Medical Science, China Medical University, 40402 Taichung, Taiwan; 7 School of Chinese Medicine, China Medical University, 40402 Taichung, Taiwan; 8 Department of Health and Nutrition Biotechnology, Asia University, 41354 Taichung, Taiwan; 9 Medical Research Center For Exosomes and Mitochondria Related Diseases, China Medical University Hospital, China Medical University, 40402 Taichung, Taiwan

## Abstract

Dysfunctional mitochondria have been shown to enhance cancer cell proliferation, reduce apoptosis, and increase chemoresistance. Chemoresistance develops in nearly all patients with colorectal cancer, leading to a decrease in the therapeutic efficacies of anticancer agents. However, the effect of dynamin-related protein 1 (Drp1)-mediated mitochondrial fission on chemoresistance in colorectal cancer is unclear. Here, we found that the release of high-mobility group box 1 protein (HMGB1) in conditioned medium from dying cells by chemotherapeutic drugs and resistant cells, which triggered Drp1 phosphorylation *via* its receptor for advanced glycation end product (RAGE). RAGE signals ERK1/2 activation to phosphorylate Drp1 at residue S616 triggerring autophagy for chemoresistance and regrowth in the surviving cancer cells. Abolishment of Drp1 phosphorylation by HMGB1 inhibitor and RAGE blocker significantly enhance sensitivity to the chemotherapeutic treatment by suppressing autophagy. Furthermore, patients with high phospho-Drp1^Ser616^ are associated with high risk on developing tumor relapse, poor 5-year disease-free survival (DFS) and 5-year overall survival (OS) after neoadjuvant chemoradiotherapy (neoCRT) treatment in locally advanced rectal cancer (LARC). Moreover, patients with RAGE-G82S polymorphism (rs2070600) are associated with high phospho-Drp1^Ser616^ within tumor microenvironment. These findings suggest that the release of HMGB1 from dying cancer cells enhances chemoresistance and regrowth via RAGE-mediated ERK/Drp1 phosphorylation.

## Introduction

Colorectal cancer (CRC) is one of the leading cause of death worldwide^1^, accounting for approximately 9.7% of all cancer cases and approximately 8.5% of cancer deaths. Currently, the major chemotherapy drugs for the treatment of CRC include oxaliplatin (OXP), 5-fluorouracil (5-FU) and irinotecan (CPT-11). However, a considerable proportion of CRC patients develop local recurrence and distant metastasis within 5 years after surgical treatment. The overall survival of CRC patients remains poor with a median of 12–18 months, and the response to chemotherapy is only 50%^2^. Moreover, most metastatic CRC patients develop resistance to OXP as the cancer progresses within 8 months^3^.

Mitochondria are organelles that provide most of the energy to nearly all cells because of their synthesis of ATP via oxidative phosphorylation. Mitochondria are key organelles for cellular homeostasis, which is regulated by the following dynamic networks: fusion and fission^4^. Fusion is mediated by Mitofusin-1 and Mitofusin-2 (Mfn1 and Mfn2) and optic atrophy 1 (Opa1) proteins located at the outer and inner mitochondrial membranes, respectively. Fission is mediated by dynamin-related protein 1 (Drp1), which is a cytosolic protein that is recruited to the surface of mitochondria during activation, leading to mitochondria fragmentation. Cumulative evidence has revealed that unbalanced mitochondrial dynamics dysregulate key cellular processes, potentially contributing to tumorigenesis^5,6^, including lung, bladder, breast, and colon cancers^7–10^. An imbalance in the expression of Drp1/Mfn is associated with excess mitochondrial fission and impaired mitochondrial fusion, which are important for the cell cycle progression^5,7^. Recently, the mitogen-activated protein kinase (MAPK) pathway has been shown to result in an increased mitochondrial fragmentation and promote tumor growth and chemoresistance via the phosphorylation of the mitochondrial protein Drp1 at serine 616 by extracellular signal-regulated kinase 2(ERK 2) in several cancers^11–14^. However, to date, the molecular mechanisms by which the dysregulated mitochondrial dynamics contribute to cancer cell survival remain unclear.

High-mobility group box 1 protein (HMGB1) is a highly conserved nuclear protein that functions as a chromatin-binding factor that bends DNA and promotes access to transcriptional protein assemblies on specific targets. In addition to its intra-nuclear role, HMGB1 functions as an extracellular signaling molecule. Released HMGB1 mediates diverse responses by binding to several receptors, including the receptor for advanced glycation end products (RAGEs) and toll-like receptors (TLR)-2 and 4, thereby triggering pleiotropic effects, such as cell proliferation, differentiation, death, inflammation, and immunity. Moreover, HMGB1 passively released from dying tumor cells after chemotherapy and radiotherapy or directly secreted from tumor cells promotes regrowth, proliferation and metastasis^15,16^. It may facilitate autophagy following cytotoxic insults for chemoresistance via its receptor RAGE through the MEK/ERK signaling pathway in colorectal cancer and lung adenocarcinoma^17–21^. Moreover, the expression of RAGE is closely associated with the invasion and metastasis of gastric cancer and colorectal cancer^22,23^. The germ-line single-nucleotide polymorphism (SNP) of RAGE with Gly82Ser (rs2070600), which are known to display increased ligand binding to enhance the downstream signaling pathway^24,25^, is associated with a significantly increased risk of several cancer types^26^. However, the mechanism and significance of HMGB1-mediated autophagy for chemoresistance remain unknown.

Here, we showed that ERK-mediated Drp1 phosphorylation is necessary for resisting chemotherapeutic cytotoxicity and reported for the first time that this mediation was associated with extracellular HMGB1 released from dying tumor cells. Extracellular HMGB1 promoted ERK activation-induced Drp1 phosphorylation via RAGE, which induced autophagy and acted upon surviving cancer cells to promote regrowth. Furthermore, patients with highly phosphorylated Drp1 proteins are associated with the increased risk on developing tumor relapse after neoadjuvant chemoradiotherapy (neoCRT) treatment in locally advanced rectal cancer (LARC). Moreover, patients with RAGE-G82S polymorphism (rs2070600) are positively associated with phospho-Drp1^Ser616^ after neoCRT treatment. Altogether, our findings showed that the HMGB1-induced Drp1-dependent mitochondrial dynamics via RAGE-ERK signaling pathway for autophagy and chemoresistance could be candidate therapeutic targets for new treatment strategies in colorectal cancer.

## Materials and methods

### Cell culture and reagents

The human colorectal cancer cell lines SW480, SW620, LoVo and LoVo^OXR^ were cultivated in RPMI 1640 growth medium supplemented with 10% fetal bovine serum (Invitrogen, California, USA) at 37 °C under a humidified atmosphere of 5% CO_2_ and 95% air.

To prepare the conditioned medium (CM), the cells were washed and incubated with a serum-free medium for 2 h when sub-confluent. The medium was discarded, and the cells were incubated with a serum-free medium again. After 48 h, the CM was harvested and centrifuged to remove debris, filtered through a 0.22 μm filter, and stored at −20 °C until use.

### Antibodies and reagents

The antibodies used in this study were as follows: anti-β-actin (sc-8432, Santa Cruz, CA, USA), anti-ERK1/2 (sc-135900, Santa Cruz, CA, USA), anti-p-ERK1/2 (sc-7383, Santa Cruz, CA, USA), anti-MFN1 (sc-50330, Santa Cruz, CA, USA), anti-RAGE (sc-365154, Santa Cruz, CA, USA and ab89911, Abcam, Cambridge, UK), anti-MFN2 (sc-100560, Santa Cruz, CA, USA), anti-OPA1(sc-393296, Santa Cruz, CA, USA), anti-phospho-Drp1^Ser616^ (#3455, Cell Signaling Technology, MA, USA), anti-phospho-Drp1 ^Ser637^ (#6319, Cell Signaling Technology, MA, USA), anti-LC3 (#3868, Cell Signaling Technology, MA, USA), anti-HMGB1 (#3935, Cell Signaling Technology, MA, USA and #651401, Biolegend, San Diego, CA, USA), and anti-p62 (#39749, Cell Signaling Technology, MA, USA). All secondary antibodies (HRP-conjugated anti-rabbit, anti-mouse and anti-goat) were purchased from Santa Cruz Biotechnology. The lentivirus carrying shRNAs against RAGE, HMGB1, Drp1, MFN1 and OPA1 were obtained from the National Core Facility for Manipulation of Gene Function by RNAi, miRNA, miRNA sponges, and CRISPR/Genomic Research Center, Academia Sinica. The recombinant HMGB1 proteins, HMGB1 inhibitor quercetin were purchased from Sigma (St. Louis, MO, USA). RAGE inhibitor FPS-ZM1 was purchased from Merck (Temecula, CA, USA).

### Western blot analysis

The conditioned medium was collected by ultrafiltration using a Microcon filter (10,000-Da cutoff; Millipore, MA, USA) to purify the secreted protein. Either the total lysates (30 μg) or subcellular fractions (10 μg) were separated via 6–12% sodium dodecyl sulfate polyacrylamide gel electrophoresis (SDS-PAGE) and transferred onto a PVDF membrane (GE Healthcare, Amersham, UK)^27^. The membranes were blocked with 5% non-fat milk and probed with specific antibodies overnight at 4 °C. Horseradish peroxidase (HRP)-conjugated secondary antibodies (1:10,000, GE Healthcare, Amersham, UK) and the Immobilon Western Chemiluminescent HRP Substrate (Millipore, MA, USA) were used. A densitometric analysis of the western blots was performed using an AlphaImager2200 digital imaging system (Digital Imaging System, CA, USA). The digital images were processed using Adobe Photoshop 7.0. Each blot was stripped using Restore Western Blot Stripping Buffer (Pierce, IO, USA) to incubate with the other antibodies. The results were assessed using Image J software (NIH, MD, USA).

### Assessment of cell growth and apoptosis, and mitochondria morphology

The cell growth was assessed using an MTT assay. Apoptosis and necrosis were assessed using the apoptosis/necrosis detection kit (Enzo Life Sciences, Plymouth Meeting, USA). According to the kit, the apoptotic and necrotic cells were labeled fluorescently by annexin V-EnzoGold and 7-AAD, respectively. The cells (2000) were observed to determine apoptosis or necrosis under a fluorescence microscope. The experiments were performed in triplicate. Lactate dehydrogenase C (LDH) and Terminal deoxynucleotidyl transferase (TdT)-mediated dUTP-biotin nick end labeling (TUNEL) were also performed.

Cells were stained with MitoTracker Red (MTR, Life Technologies #M7512). Cells were stained with the 100 nM MTR in completed medium for 30 min at 37 °C, after which the media was replaced for imaging.

### Animal model

Athymic nude BALB/c mice (male, 4-weeks-old) were maintained according to the institutional guidelines approved by the China Medical University Institutional Animal Care and Use Committee. The serum HMGB1 level was examined on day 9. LoVo, LoVo^OXR^, SW480-vector, SW480-Drp1^WT^, SW480-Drp1^S616E^ (1 × 10^6^ cells/mouse) were suspended in Matrigel matrix with 50% serum-free DMEM, and injected subcutaneously into the flank of each mouse. The tumor volume, body weight and survival time were measured at various time intervals throughout the study. The tumor volumes were calculated according to the formula (width^2^ × length)/2. The mice were sacrificed at the termination of the experiments, and the serum and tumor tissues from representative mice were collected for lysis, subjected to the immunoblotting analysis and stained by immunohistochemistry.

### Enzyme-linked immunosorbent assay (ELISA)

The mouse blood was rapidly centrifuged at 500 × *g* for 4 min at 4 °C. The supernatant was used as the serum. The HMGB1 in the serum was measured using ELISA kit (Shinotest, Tokyo, Japan, and Abnova, Taipei City, Taiwan). ELISA was performed according to manufacturer’s instructions. The experiment was repeated three times.

### Patient selection, clinical staging, treatment, and pathological evaluation

Between 2006 and 2014, patients with locally advanced rectal cancer were treated at the China Medical University Hospital. Patients with biopsy-proven, locally advanced rectal cancer [cT3-4 or cN+ by endorectal ultrasonography (EUS), computed tomography (CT), or magnetic resonance imaging (MRI)] who were treated with preoperative chemoradiotherapy followed by radical resection at the China Medical University Hospital were the study cohorts. The study was reviewed and approved by the Internal Review Board (IRB) of the China Medical University Hospital [Protocol number: CMUH105-REC2-072]. Patients with concurrent distant metastasis or concurrent inflammatory bowel disease, hereditary colorectal cancer syndromes, concurrent malignancy, emergent surgery, prior history of radiotherapy to the pelvis, or prior history of malignancy were excluded. The tumors were staged according to the American Joint Committee on Cancer (AJCC) staging system.

The patients were treated with chemoradiotherapy at a median radiotherapy dose of 50.4 Gy and concurrent fluoropyrimidine-based chemotherapy (mainly single-agent infusional capecitabine). The age ranged from 31 to 90 years. Surgery, including low anterior resection, proctectomy with coloanal reconstruction, abdominoperineal resection, or multivisceral rectal resection using total mesorectal excision principles, was performed 6–8 weeks after the completion of chemoradiotherapy. Adjuvant chemotherapy was recommended for patients with metastatic lymph node(s) in the surgical specimen and consisted of infusional fluorouracil or capecitabine for a period of 4–6 months.

The pathological staging of the resected specimen was performed after resection in accordance with the guidelines of the College of American Pathologists, and the histopathological diagnosis was performed by pathologists specializing in gastrointestinal cancer. A pathological complete response (pCR) was defined as the absence of viable adenocarcinoma cells in the surgical specimens (ypT0N0). The complete response (cCR) classification included specimens with acellular mucin pools without viable tumor cells.

### Construction of tissue microarray (TMA)

Tissue microarrays were constructed from biopsies from 106 pair-matched pre-neoCRT and post-neoCRT rectal cancer patients (Table S1). One hundred and six pre-neoCRT biopsies and 93 [13 tumor regression grade 4 (TRG 4)] post-neoCRT surgical specimens were evaluated. The areas of the tumor were marked on hematoxylin & eosin (H & E) stained slides. The corresponding area on the matching paraffin block (donor block) was then identified and marked. We used the AutoTiss 10C system (EverBio Technology, Inc., Taipei, Taiwan) to remove the tissue core from these areas on the donor blocks, which were placed into the recipient block in a precisely arrayed fashion. The diameter of the punches was 2 mm. A maximum of 60 punches were placed on one single block. The sample sections (cut via a microtome) were then mounted on capillary-gap slides (Dako, Hamburg, Germany) and baked overnight^28^.

### Immunohistochemistry

The 3μm-thick TMA slides were individually stained with horseradish peroxidase-conjugated avidin biotin complex (ABC) from the Vectastain Elite ABC Kit (Vector Laboratories, Burlingame, CA) and NovaRed chromogen (Vector Laboratories, Burlingame, CA) and counterstained with hematoxylin. Positive staining for Drp1 and p-Drp1 was detected in the cytoplasm. The Drp1 or p-Drp1 staining patterns were evaluated in the sections according to the proportion of positively stained tumor cells under a microscope (OLYMPUS BX53, Tokyo, Japan). The intensity of the staining was evaluated, and five fields were used in the evaluation^29^. The pathologists estimated the number of immunopositive cells. The staining intensity was scored as follows: a score of 0 indicated no color; light red was scored as 1; purple-red was scored as 2; and dark-red was scored as 3. The percentage of positive cells was scored as follows: a score of 0 was assigned when the positive tumor cell proportion was 10%; 1 point was assigned when the positive cell proportion ranged from 10 to 25%; 2 points were assigned when the positive cell proportion ranged from 26 to 50%; and 3 points were assigned when the positive cell proportion ranged from 51% to 100. The sum of the two scores served as the staining score. Using score of 3 as a cutoff, the immunostains were defined as “low” for scores from 0 to 3 and as “high” for scores from 4 to 9^30^.

### RAGE germ-line single-nucleotide polymorphism (SNP) analysis

Genomic DNA from rectal cancer patients was extracted from the FFPE tissue using a QuickExtract™ FFPE DNA Extraction Kit (Epicenter, WI, USA). For the SNP genotyping, 10 ng to total genomic DNA were used for the PCR amplification using the following specific primers: 5′-RAGE G82S (5′- ATTTGGATCCCCGTCACTCTG -3′) and Biotin-3′- RAGE G82S (5′- GCCTGGCACCGGAAAATC -3′).

The biotinylated PCR products were immobilized into streptavidin-coated super paramagnetic Dynabeads M280-streptavidin. The sequencing primers and RAGE-G82S-Seq (5′-CGTGTCCTTCCCAAC-3′) were annealed to the DNA templates according to the manufacturer’s instructions (PSQk 96MA instrument, Qiagen).

### Statistical analysis

The statistical analysis was performed using SAS statistical software, version PC 9.4 (SAS Institute, Cary, NC, USA). A two-sided *p*-value at a significance level of 0.05 was reported for all tests. The between-group comparisons were performed using Student’s *t*-test, Pearson chi-square test and Fisher’s exact test. A Cox regression analysis was performed to estimate the hazard ratios (HRs) and 95% confidence intervals (CIs) for the univariate and multivariate models. The influential factors affecting the survival rate of the patients with rectal cancer were adjusted in the Cox models—including sex (female versus male), age (<65 vs. ≥65), TRG (3–4 vs. 1–2), clinical response (good response vs. poor response), and pN stage (positive vs. negative). The Kaplan–Meier estimation method was used to assess the five-year overall survival and disease-free survival. The survival time was defined as the time from surgery to death.

## Results

### Mitochondrial fission protein Drp1 is hyperactivated upon cytotoxic insults and promotes chemoresistance

**Fig. 1:**
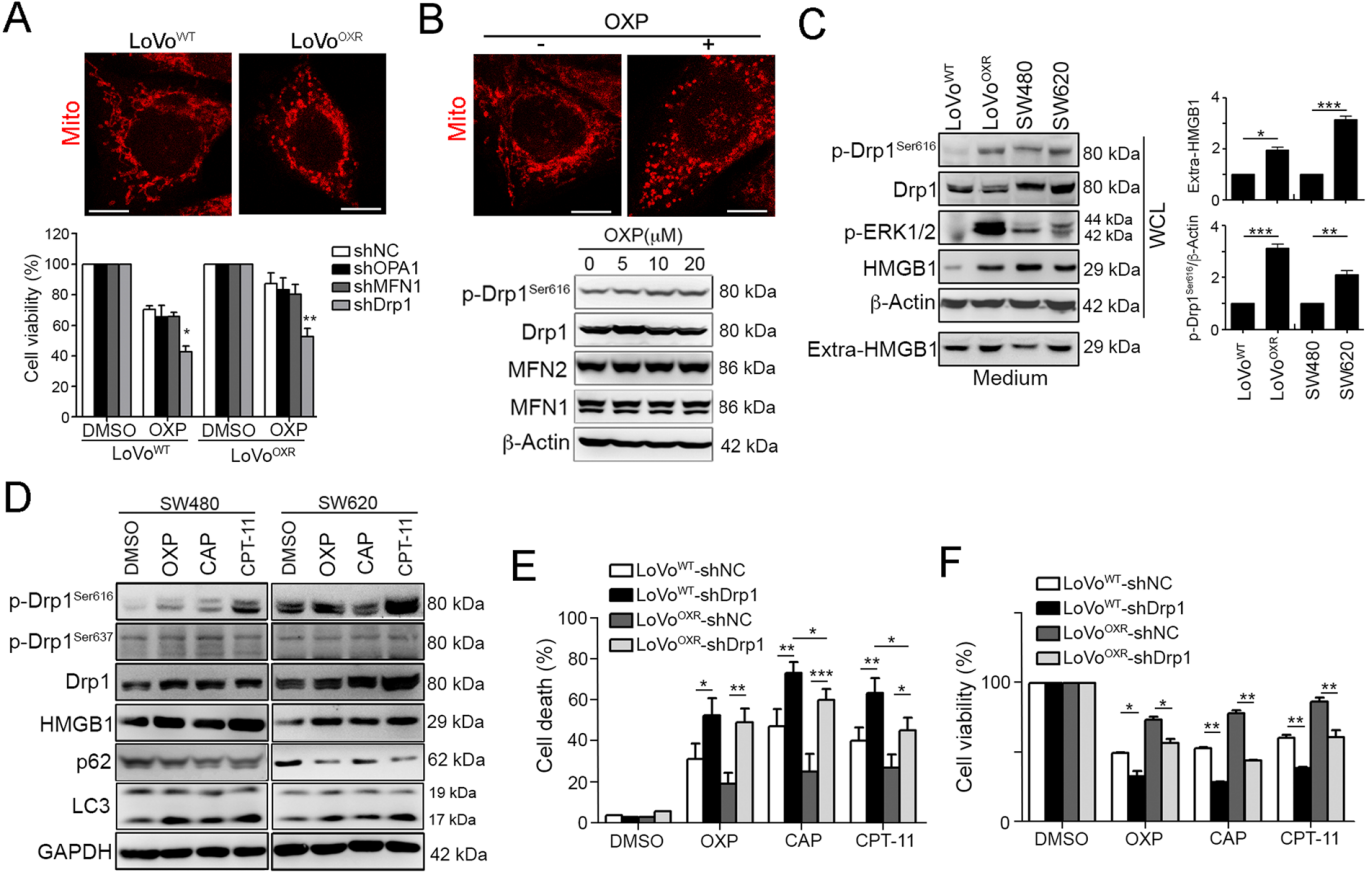
Drp1 is highly phosphorylated upon cytotoxic insults to promote chemoresistance. **a** LoVo^WT^ and LoVo^OXR^ were infected by lentivirus carrying shRNA against OPA1, Drp1, and MFN1. These cells were treated with oxaliplatin (OXP, 10 μM) for 48 h. The cell viability was analyzed using an MTT assay (*n* = 3). *p < 0.01 and **p < 0.01. **b** Mitochondria morphology was observed by MitoTracker Red staining. Scale bar = 10 μm. **c** LoVo^WT^ cells were treated with diverse concentrations of OXP for 24 h. Cell lysates were analyzed by immunoblotting. Mitochondria morphology was observed by MitoTracker Red staining. Scale bar = 10 μm. **d** Cell lysate and medium were analyzed by immunoblotting. Quantification of these results is shown (*n* = 3). *p < 0.05, **p < 0.01 and ***p < 0.001. **e** SW480 and SW620 cells were treated with OXP (10 μM), CAP (10 μM), or CPT-11 (5 μM) for 24 h. Cell lysates were analyzed by immunoblotting. **f** LoVo and LoVo^OxR^ cells with shNC and shDrp1 were treated with OXP (10 μM), CAP (10 μM), or CPT-11 (10 μM) for 48 h. Cell death was analyzed using an AnnexinV/PI assay (*n* = 3). *p < 0.05, **p < 0.01 and, ***p < 0.001. **g** LoVo and LoVo^OxR^ cells carrying shNC and shDrp1 were treated with OXP (10 μM Oxaliplatin), CAP (10 μM), or CPT-11 (5 μM) for 48 h. The cell viability was analyzed using an MTT assay (*n* = 3). *p < 0.05 and **p < 0.01

By comparing the mitochondria dynamics between LoVo and oxaliplatin (OXP)-resistant LoVo cells, we found that mitochondria morphology was dramatic changed in LoVo^OXR^ cells (Fig. 1a). We then aim to identify the mitochondrial proteins that are involved in chemoresistance in CRC. By analyzing the LoVo^WT^ and LoVo^OXR^ cell lines, we found that the knockdown of the mitochondrial fusion proteins OPA1 and MFN1 did not sensitize the LoVo^OXR^ cells to oxaliplatin (Fig. 1a). The knockdown efficiency was shown in Fig. S1A. Only knockdown of the mitochondrial fission protein Drp1 sensitized both the LoVo^WT^ and LoVo^OxR^ cells to OXP, suggesting that Drp1 might influence chemosensitivity in CRC (Fig. 1a). We then investigated the Drp1 expression and phosphorylated status following exposure to chemotherapeutic drugs. A strikingly elevated phosphorylation of activated Drp1 (Ser616) was observed following the chemotherapeutic drug OXP treatment (Fig. 1b) But there is no variation on mitochondrial proteins MFN1 and MFN2 upon OXP treatment, suggesting that only Drp 1 may participate in the chemoresistance via phosphorylation status. We then evaluated the phosphorylation status of Drp1 in well-characterized chemoresistant and metastatic colorectal cancer cell lines. By immunoblotting results, we found that Drp1 S616 phosphorylation were also significantly upregulated in LoVo^OXR^ and SW620 cell lines. Moreover, the high levels of extracellular HMGB1 and phosphorylation of ERK1/2 were found in the OXP-resistant LoVo^OXR^ (compared to LoVo^WT^) and metastatic SW620 (compared to SW480, Fig. 1c).

Recent studies have shown that mitochondrial fission via Drp1 significantly promoted cell survival by increasing autophagy, which promoted resistance to apoptosis and induced chemoresistance^10,11^. The immunoblotting results showed that Drp1 and phospho-Drp1^Ser616^ were remarkably upregulated following diverse chemotherapeutic drug treatments, such as OXP, capecitabine (CAP, the pro-drug of 5-FU) and irinotecan (CPT-11) (Fig. 1d, Fig. S1B, C). Moreover, the amount of the mitochondrial autophagy adapter protein p62 was decreased and LC3-II was increased in response to chemotherapeutic drugs, implying that the chemotherapeutic drugs may induce autophagy to promote chemoresistance in CRC (Fig. 1d). Anticancer agents OXP, CAP, and CPT-11 also significantly promoted the HMGB1 expression in CRC (Fig. 1d).

To confirm the Drp1 phosphorylation promotes chemoresistance, we silenced Drp1 by lentivirus carrying shRNA against Drp1. Indeed, knockdown of Drp1 increased the chemosensitivity of LoVo^OXR^ cells to chemotherapeutic drugs, promoting the cell death (Fig. 1e) and decreasing cell viability (Fig. 1f). Moreover, treatment with Drp1 inhibitor Mdivi-1 also increased the sensitivity to OXP either LoVo^WT^ or LoVo^OXR^ (Fig. S1D). These results indicated that Drp1-mediated mitochondrial fission notably promoted chemoresistance in CRC cells.

### Highly phosphorylated Drp1 may promote chemoresistant tumor growth through HMGB1/RAGE signaling pathway

**Fig. 2:**
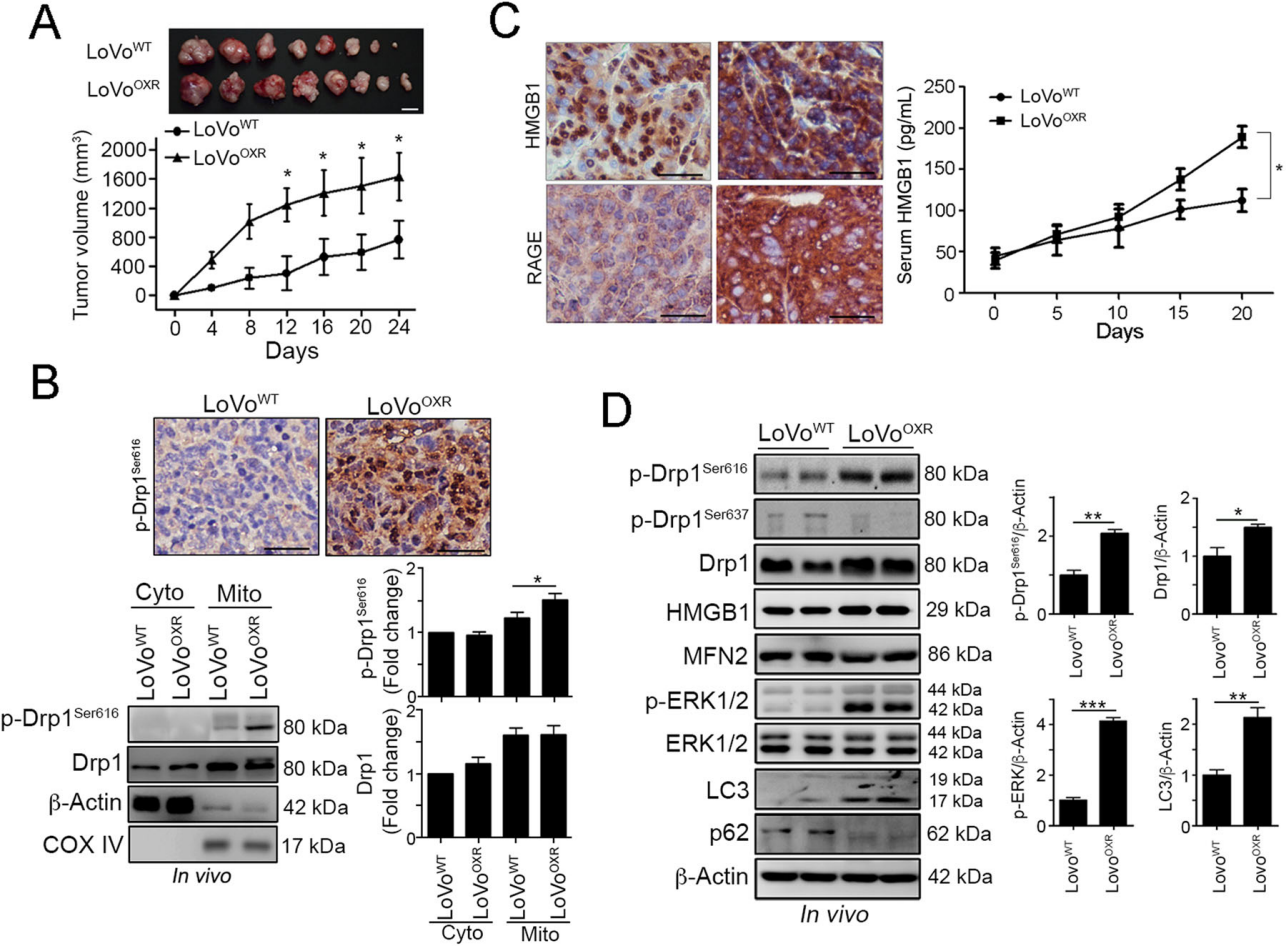
Extracellular HMGB1 and phosphorylated Drp1 were highly upregulated in the chemoresistant CRC in vivo. **a** Athymic nude BALB/c mice were subcutaneously inoculated with LoVo^WT^ and LoVo^OXR^ cells into the flank of each mouse. (1 × 10^6^ cells/ mice, *n* = 6). The tumor volume was measured at various time intervals throughout the study. Quantification of these results is shown (*n* = 6). *p < 0.05. **b** Xenografted tumors from representative mice were stained for phospho-Drp1^Ser616^ by immunohistochemistry. Bar: 10 μm. Xenografted tumors from representative mice were homogenized and isolated for the cytoplasmic and mitochondrial fraction analysis. Then, these fractions were analyzed by immunoblotting. Quantification of these results is shown (*n* = 3). *p < 0.05 and **p < 0.01. **c** Xenografted tumors from LoVo^WT^- and LoVo^OXR^-injected mice were analyzed by immunohistochemistry. Bar: 10 μm. Serum from LoVo-injected and LoVo^OxR^-injected mice was analyzed by ELISA. Quantification of these results is shown (*n* = 6). *p < 0.05. **d** Xenografted tumors from representative mice were collected for lysis and analyzed by immunoblotting. Quantification of these results is shown (*n* = 3). *p < 0.05 and **p < 0.01. These data were obtained from three independent experiments, and the values represent the means ± S.D

To verify the role of Drp1 on chemoresistant CRC in vivo, we inoculated athymic nu/nu mice with LoVo^WT^ and LoVo^OXR^. Tumors derived from LoVo^WT^ grew more slowly compared with those derived from LoVo^OXR^ (Fig. 2a). We observed that the LoVo^OXR^ showed hyperactivated Drp1 levels in vivo (Fig. 2a). Moreover, the immunoblotting results showed that the phosphorylated Drp1^Ser616^ protein level was highly recruited to the mitochondrial fraction in the tumor derived from LoVo^OXR^ cells (Fig. 2b). These results show that Drp1 is important for modulating chemoresistance *via* mitochondria fission in CRC cells in vivo.

Moreover, immunohistochemistry analysis showed that the level of RAGE and the distribution of HMGB1 in LoVo^OXR^ group was altered. Cytosolic translocation of HMGB1 was significantly increased in LoVo^OXR^ group, suggesting that HMGB1 was translocated from nucleus into cytoplasm, and then secreted into plasma. Consistent with this observation, high serum HMGB1 was detected in LoVo^OXR^ group (Fig. 2c). Moreover, the results of immunohistochemistry also showed that RAGE, the receptor of HMGB1, was greatly upregulated in LoVo^OXR^ group (Fig. 2c). Immunoblotting results from resected tumor tissues also revealed high HMGB1 level, active ERK1/2 and phospho-Drp1^S616^ in LoVo^OXR^ group (Fig. 2d). We also observed an increased level of LC3-II expression and decrease p62 level in LoVo^OXR^ tumor comparison with control LoVo^WT^ tumor (Fig. 2d). Moreover, the mitochondrial fusion protein MFN2 was not influenced between these two tumors. These results implied the critical role of HMGB1 in modulating the chemosensitivity via ERK-mediated Drp1 CRC in vitro and in vivo.

### HMGB1 triggered ERK-mediated Drp1 phosphorylation via RAGE in CRC

**Fig. 3:**
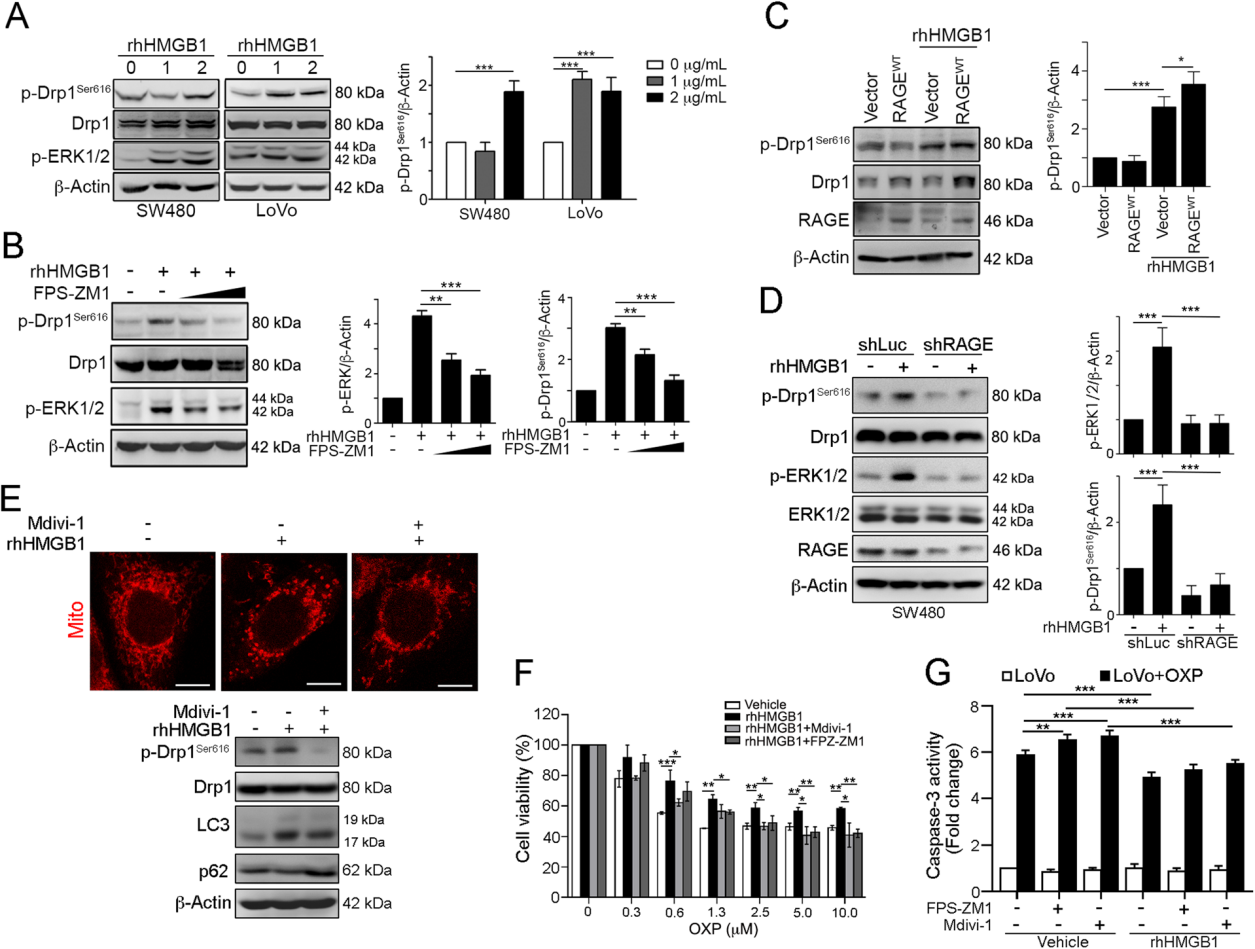
HMGB1 triggered ERK-mediated Drp1 phosphorylation via RAGE in CRC. **a** SW480 and LoVo cells were treated with the recombinant human HMGB1 protein (0, 1, and 2 μg/mL) for 24 h. Cell lysate was analyzed by immunoblotting. Quantification of these results is shown (*n* = 3). ***p < 0.001. **b** SW480 cells were treated with the recombinant human HMGB1 protein (1 μg/mL) and RAGE inhibitor (10 μM FPS-ZM1) for 24 h. Cell lysate was analyzed by immunoblotting. Quantification of these results is shown (*n* = 3). **p < 0.01 and ***p < 0.001. **c** SW480 cells were transfected with the pcDNA3.1-vector, pcDNA3.1-wtRAGE for 24 h and then treated with the recombinant human HMGB1 protein (1 μg/mL) for 24 h. Cell lysate was analyzed by immunoblotting. Quantification of these results is shown (*n* = 3). **p < 0.01. **d** SW480 cells were silenced with lentivirus carrying shRNA again RAGE, and then treated with the recombinant human HMGB1 protein (1 μg/mL) for 24 h. Cell lysate was analyzed by immunoblotting. **e** LoVo cells were treated with recombinant human HMGB1 (1 μg/mL) for 24 h. Cell lysate was analyzed by immunoblotting. Quantification of these results is shown (*n* = 3). ***p < 0.001. Mitochondria morphology was observed by MitoTracker Red staining. Scale bar = 10 μm. **f** LoVo cells were treated with RAGE inhibitor (10 μM FPS-ZM1), Drp1 inhibitor (50 μM Mdivi-1) and recombinant human HMGB1 (1 μg/mL) for 1 h prior to addition of different dosages of OXP for 48 h. The cell viability was analyzed using an MTT assay (*n* = 3). *p < 0.05. **g** LoVo cells were treated with RAGE inhibitor (10 μM FPS-ZM1), Drp1 inhibitor (50 μM Mdivi-1) and recombinant human HMGB1 (1 μg/mL) for 1 h prior to addition of OXP (10 μM) for 48 h. The caspase-3 activity was analyzed (*n* = 3). **p < 0.01. These data were obtained from three independent experiments, and the values represent the means ± S.D

To confirm the relationship between HMGB1 and Drp1 phosphorylation, we treated the CRC cells with recombinant HMGB1 and observe its influence on ERK/Drp1 signaling. We found that ERK and Drp1 were activated by the HMGB1 recombinant protein in both SW480 and LoVo cell lines (Fig. 3a). Inhibition of RAGE by FPS-ZM1^31^ blocked the HMGB1-induced Drp1 phosphorylation and ERK1/2 activation in dose-dependent manner (Fig. 3b). Furthermore, enforced expression of wt-RAGE sensitized the HMGB1-induced Drp1 phosphorylation (Fig. 3c). Similarly, knockdown of RAGE by lentivirus-based shRNA significantly alleviated HMGB1-induced Drp1 phosphorylation and ERK1/2 activation (Fig. 3d).

Furthermore, addition of HMGB1 dramatically influences the mitochondria morphology and increases conversion of LC3-I to LC3-II and decreases p62 level (Fig. 3e). Treatment with Drp1 inhibitor Mdivi-1, which is a small molecular inhibitor of Drp1^32^, mitigates HMGB1-induced Drp1 phosphorylation, mitochondria morphology alteration, LC3 conversion and p62 levels decrease.

To identify whether HMGB1 was responsible for chemoresistance via Drp1 phosphorylation, we pre-treated with recombinant HMGB1 for 1 h prior to addition of OXP (Fig. 3f). Pre-incubation of HMGB1 significantly alleviated OXP-induced cell viability decrease. But blockade of RAGE by FPS-ZM1 or inhibition of Drp1 by Mdivi-1 neutralized the OXP-induced cell death. Addition of HMGB1 prior to OXP treatment also reduced the OXP-induced caspase-3 activity. However, the caspase-3 activity was decreased by FPS-ZM1 and Mdivi-1, suggesting that HMGB1 is required for chemoresistance to OXP via Drp1. Taken together, these results indicated that the extracellular HMGB1 promotes ERK activation-mediated Drp1 phosphorylation resulting in tumor growth and chemoresistance via RAGE.

### Secretion of HMGB1 upon cytotoxic insults promotes Drp1 phosphorylation for chemoresistance via autophagy

**Fig. 4:**
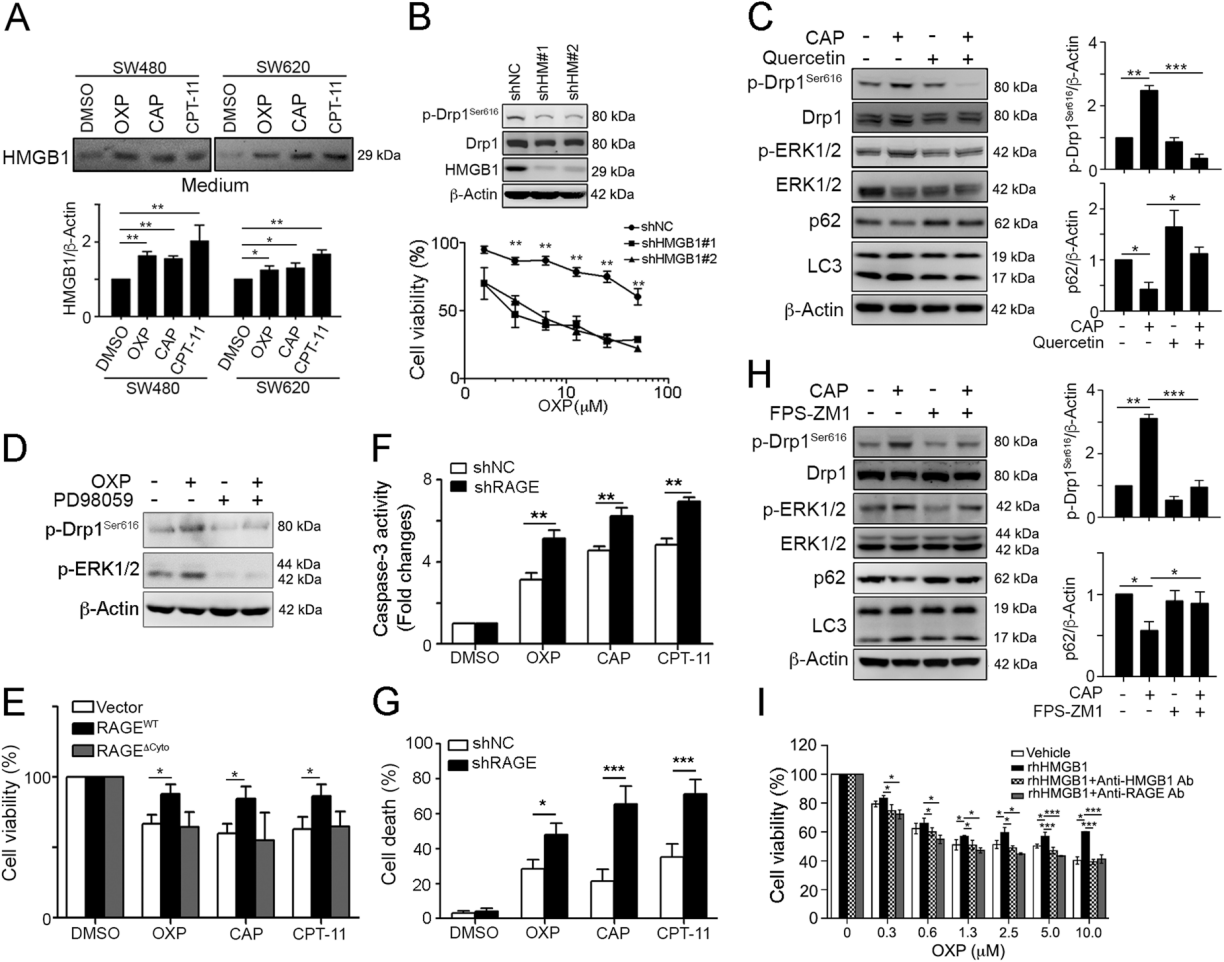
Extracellular HMGB1 by chemotherapy triggered ERK-mediated Drp1 phosphorylation for autophagy and chemoresistance via RAGE in CRC. **a** SW480 and SW620 cells were treated with OXP (10 μM), CAP (10 μM), or CPT-11 (5 μM) for 24 h. Conditioned medium were analyzed by immunoblotting. Quantification of these results is shown (*n* = 3). *p < 0.05, **p < 0.01 and ***p < 0.001. **b** SW480 cells carrying shNC, shHMGB1^#1^ and shHMGB1^#2^ were treated with various concentration of oxaliplatin for 48 h. The cell viability was analyzed using an MTT assay (*n* = 3). **p < 0.01. **c** SW480 cells were treated with CAP (10 μM) and HMGB1 inhibitor (Quercetin, 50 μM) for 24 h. Cell lysate was analyzed by immunoblotting. Quantification of these results is shown (*n* = 3). **p < 0.01 and ***p < 0.001. **d** SW480 cells were treated with OXP (10 μM) and PD98059 (10 μM) for 24 h. Cell lysate was analyzed by immunoblotting. **e** SW480 cells were transfected with the pcDNA3.1-vector, pcDNA3.1-wtRAGE and pcDNA3.1-RAGEΔCyto for 24 h and then treated with OXP (10 μM), CAP (10 μM), or CPT-11 (5 μM) for 48 h. The cell viability was analyzed using an MTT assay (*n* = 3). *p < 0.05. **f** SW480 cells carrying shNC and shRAGE were treated with OXP (10 μM Oxaliplatin), CAP (10 μM), or CPT-11 (5 μM) for 48 h. The caspase-3 activity was analyzed (*n* = 3). **p < 0.01. **g** SW480 cells carrying shNC and shRAGE were treated with OXP (10 μM), CAP (10 μM), or CPT-11 (5 μM) for 48 h. Cell death was analyzed using an AnnexinV/PI assay (*n* = 3). *p < 0.05 and ***p < 0.001. **h** SW480 cells were treated with CAP (10 μM) and RAGE inhibitor (FPS-ZM1, 10 μM) for 24 h. Cell lysate was analyzed by immunoblotting. Quantification of these results is shown (*n* = 3). **p < 0.01 and ***p < 0.001. **i** LoVo cells were pre-incubated with anti-RAGE or anti-HMGB1 neutralizing antibodies (2.5 μg/mL) for 30 min prior to addition of recombinant HMGB1 and OXP for 48 h. The cell viability was analyzed using an MTT assay (*n* = 3). *p < 0.05. These data were obtained from three independent experiments, and the values represent the means ± S.D

To verify the HMGB1 secreted from dying cells can promote Drp1 phosphorylation, we challenges with diverse chemotherapeutic drugs and collected the medium for analysis. Indeed, extracellular HMGB1 secreted from dying tumor cells was detected in the medium following treatment with these anticancer agents (Fig. 4a). We then silenced HMGB1 using a lentivirus carrying shRNA against HMGB1 to observe the impact on Drp1 phosphorylation. As expected, the knockdown of HMGB1 clearly decreased phosphor-Drp1^Ser616^ (Fig. 4b). The silencing of HMGB1 also enhanced the sensitivity of colorectal cancer cells to OXP (Fig. 4b). Notably, the inhibition of HMGB1 secretion by quercetin^33,34^ significantly suppressed the CAP-induced ERK activation and Drp1 phosphorylation (Fig. 4c). The decrease in p62 and increase in LC3-II were also attenuated by quercetin, suggesting that CAP-induced autophagy was decreased as HMGB1/RAGE signaling was blocked. Moreover, blockade of MEK/ERK by MEK inhibitor (PD98059) significantly decreased Drp1 phosphorylation (Fig. 4d). These results suggested that the high level of HMGB1 secreted from dying and resistant cancer cells during chemotherapy may trigger Drp1 phosphorylation via RAGE/ERK.

Moreover, overexpression of the wild-type RAGE increased the cell viability following the anticancer agent treatment (Fig. 4e). The dominant-negative of RAGE (RAGE-ΔCyto) had no influence on the cell viability following the cytotoxic insults (Fig. 4e). Consistent with these results, knockdown of RAGE increased caspase-3 activity (Fig. 4f) and cell death (Fig. 4g) following the chemotherapeutic drug treatments. Notably, the blockage of RAGE by the pharmacological inhibitor FPS-ZM1 also significantly suppressed the CAP-induced ERK activation and Drp1 phosphorylation (Fig. 4h). The decrease in p62 and increase in LC3-II were also attenuated by FPS-ZM1. Moreover, pretreatment with anti-RAGE or anti-HMGB1 neutralizing antibodies for 30 min prior to addition of recombinant HMGB1 and OXP significantly mitigated HMGB1-induced chemoresistance (Fig. 4i). Taken together, these results indicated that secretion of HMGB1 from dying tumor cells promotes ERK-mediated Drp1 phosphorylation, which contributes to autophagy and chemoresistance following chemotherapeutic treatment.

### Inhibition of Drp1 alleviated chemotherapeutic drugs-induced autophagy and chemoresistance in vitro and in vivo

**Fig. 5:**
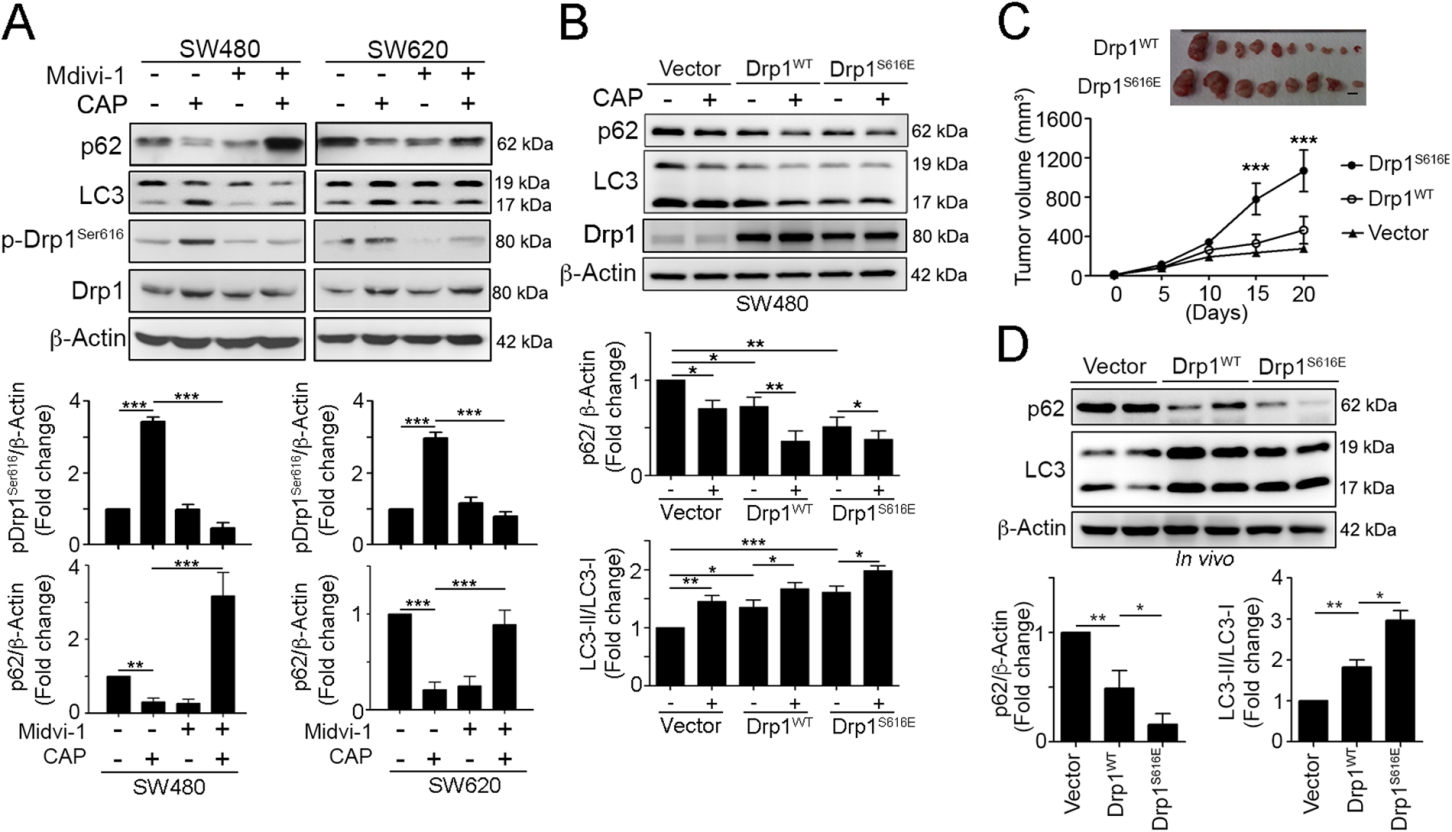
Hyperactivated Drp1 is required for autophagy turnover and tumor growth of CRC in vivo. **a** SW480 and SW620 cells were treated with CAP (10 μM) and a Drp1 inhibitor (50 μM Mdivi-1) for 24 h. Cell lysate were analyzed by immunoblotting. **b** SW480 cells were transfected with a GFP vector, GFP-wtDrp1 or GFP-Drp1^S616E^ for 24 h and then treated with CAP (10 μM Capecitabine) for 24 h. Cell lysate was analyzed by immunoblotting. Quantification of these results is shown (*n* = 3). *p < 0.05, **p < 0.01, and ***p < 0.001. **c** 1 × 10^6^ cells were injected subcutaneously into the flank of each mouse. Tumor volume, body weight and survival time were measured at various time intervals throughout the study. Quantification of these results is shown (*n* = 5). **p < 0.01. **d** Xenografted tumors from representative mice were collected for lysis and analyzed by immunoblotting. Quantification of these results is shown (*n* = 3). *p < 0.05 and **p < 0.01. These data were obtained from three independent experiments, and the values represent the means ± S.D

Consistent with previous results, we found that Drp1 inhibitor Mdivi-1 significantly alleviated the CAP-induced Drp1 phosphorylation, conversion of LC3 I to LC3II and decrease in p62 (Fig. 5a). To determine Drp1 phosphorylation is critical for drug resistance in tumor cells, we constructed a gain-of-function Drp1 containing both S616E (to mimic activating phosphorylation) and S637A (to block inhibitory phosphorylation) mutations. Enforced expression of Drp1^S616E^ accelerated the CAP-induced p62 decrease and LC3-II conversion (Fig. 5b). These results demonstrate that hyperactivated Drp1 is essential for chemoresistance via mitochondrial fission and autophagy.

To further explore the effect of the mitochondrial dynamics on cell survival in CRC cells, we inoculated athymic nude BALB/c mice with SW480 tumor cells that stably expressed hyperactivated Drp1^S616E^ or wild-type Drp1. The growth of the Drp1^S616E^ tumor cells was significantly faster than that of the wild-type Drp1–transfected tumors (Fig. 5c). We observed that increased autophagy in the resected tumor transfected with wild-type Drp1 and Drp1^S616E^ in vivo (Fig. 5d). The level of p62 was dramatically decreased and the ratio of LC3-II/I was increased. But the Drp1^S616E^-transfected tumor cells displayed more autophagy (Fig. 5d). These results showed that Drp1-mediated mitochondrial fission notably promoted autophagy and chemoresistance in CRC cells.

### Hyperactivated Drp-1 within tumor microenvironment is associated with poor 5-years disease-free survival (DFS) and 5-year overall survival in LARC patients

**Fig. 6:**
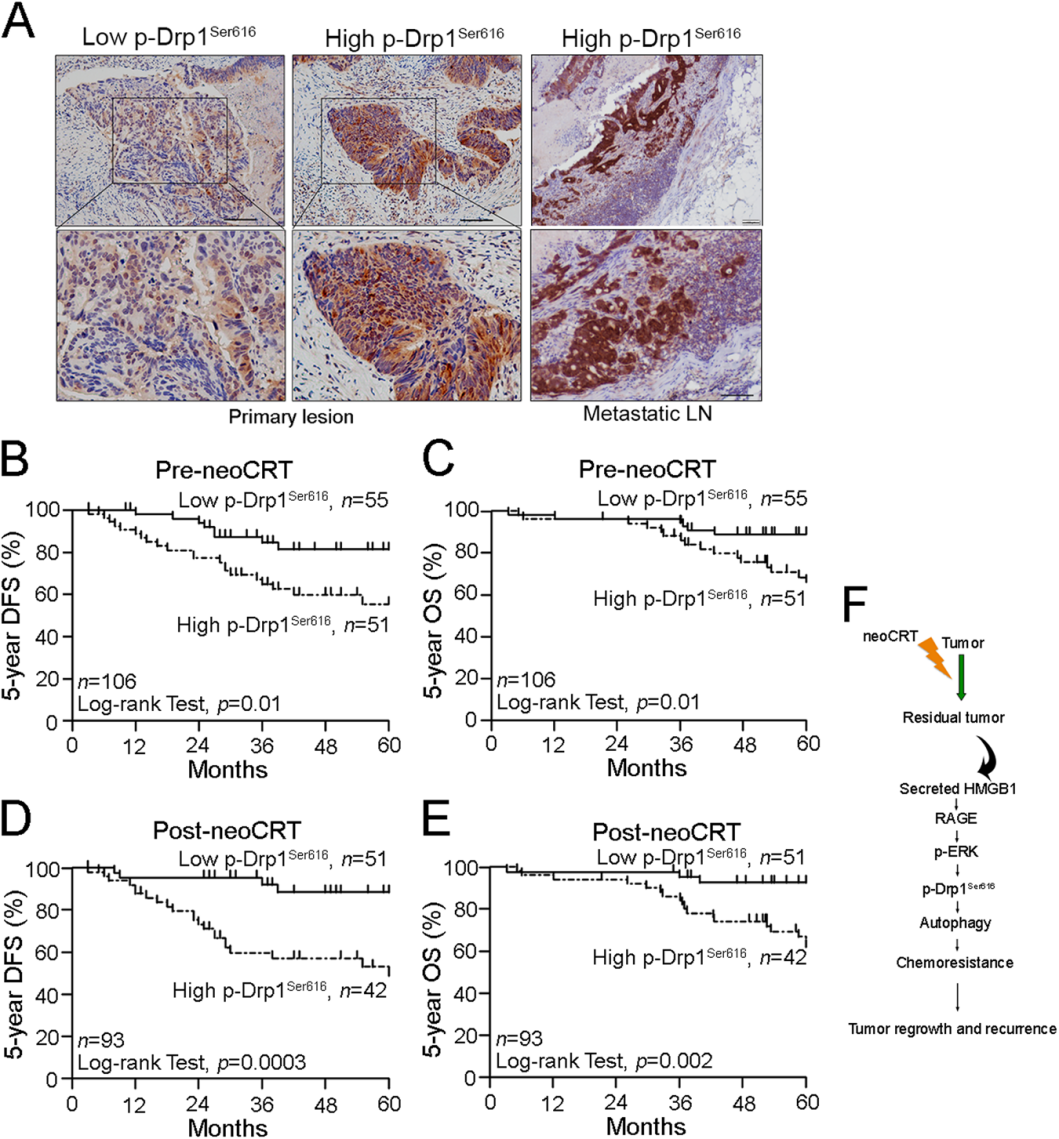
Hyperactivated Drp1 is associated with poor 5-year DFS and 5-year OS tumor growth for advanced metastasis in CRC. **a** The expression of phospho-Drp1^S616^ in the pre-neoCRT biopsies and metastatic lymph nodes. **b** High phospho-Drp1^S616^ within the tumor microenvironment of pre-neoCRT biopsies was associated with a poor 5-year DFS. **c** High phospho-Drp1^S616^ within the tumor microenvironment of pre-neoCRT biopsies was associated with a poor 5-year OS. **d** High phospho-Drp1^S616^ within the tumor microenvironment of post-neoCRT surgical tissues was associated with a poor 5-year DFS and 5-year OS. **e** High phospho-Drp1^S616^ within the tumor microenvironment of post-neoCRT surgical tissues was associated with a poor 5-year OS. **f** The proposed mechanism of HMGB1-mediated Drp1 phosphorylation *via* RAGE-ERK1/2 signaling to promote chemoresistance in colorectal cancer

To determine the clinical relevance of these findings, we performed a combination of tissue analysis studies. We performed an immunohistochemistry analysis of Drp1 and phospho-Drp1^Ser616^ on 106 pair-matched pre-neoCRT biopsies and post-neoCRT surgical tissues in LARC patients using a tissue microarray (TMA, Fig. 6a). These patients were received the preoperative neoadjuvant chemoradiotherapy (neoCRT). The median dose of radiation was 50.4 Gy in 28 fractions. The concurrent chemotherapy was fluorouracil-based in 42% of the patients and capecitabine (CAP) in 58% of the patients. After neoCRT treatment, 12% (13/106) of the patients had a complete response, while 82% (93/106) of the patients had a partial response based on the tumor regression grade (TRG). The clinicopathological characteristics are presented in Table S1.

Patients with high level of Drp1 have no significant correlation with these parameters. But patients with high level of phospho-Drp1^Ser616^ had high risk on tumor relapse within 5 years (*p* = 0.0073) (Table S1). Moreover, patients with high phospho-Drp1^Ser616^ showed increased risk on poor 5-years disease-free survival (DFS, *p* = 0.01, Fig. 6b) and 5-years overall survival (OS, *p* = 0.01, Fig. 6c) in LARC before neoCRT treatment (Table 1).

**Table 1:** Clinicopathologic parameters with 5-year DFS and 5-year OS in pre-neoCRT biopsy of LARC (*n* = 106)

Variable	All patients (*n* = 106)				
	Total cases	DFS		5-years OS	
		5-years (%)	*p* value	5-years (%)	*p* value
	106	71		73	
*cN stage*			0.37		0.65
Negative	50	76		82	
Positive	56	66		78	
*pN stage*			0.05*		0.02*
Negative	74	77		88	
Positive	32	59		66	
*Clinical response*			0.78		0.07
Good response	56	71		85	
Poor response	50	70		72	
*TRG*			0.37		0.06
Good response	74	73		84	
Poor response	32	65		68	
*Drp1(pre-neoCRT)*			0.78		0.77
Low	61	73		80	
High	45	69		78	
*p-Drp1* ^*Ser616*^ *(pre-neoCRT)*			0.01*		0.01*
Low	55	82		89	
High	51	59		69	

cN stage: positive (Stage 1 + 2) vs. negative (Stage 0); pN stage: positive (Stage 1a + 1b + 2) vs. negative (Stage 0 + X); Clinical response: good response (complete response and partial response) vs. poor response (stable disease and progression disease); TRG: good response (TRG 3–4) vs. poor response (TRG 1–2); Drp1 and p-Drp1: high (grade 2 + 3) vs. low (grade 0 + 1). Kalpan–Meier method was used for survival analysis

*SE* standard error

Furthermore, in the post-neoCRT surgical specimens (*n* = 93), we found that the patients with the high phospho-Drp1^Ser616^ still showed high risk on poor DFS (*p* = 0.0003, Fig. 6d) and 5-year OS (*p* = 0.002, Fig. 6e) in LARC. These results indicated that phospho-Drp1^Ser616^ was significantly associated with the risk of developing tumor relapse after neoCRT treatment, suggesting that phospho-Drp1^Ser616^ contributes to treatment resistance and metastasis in LARC.

### Prognostic relevance of hyperactivated Drp-1 in LARC patients receiving neoadjuvant chemoradiotherapy (neoCRT) treatment

In the univariate analysis, the patients with high level of phospho-Drp1^Ser616^ had an increased risk on poor 5-year OS (HR = 3.164, 95% CI 1.238–8.089, *p* = 0.016, Table 2). In the multivariate analysis, the patients with high phospho-Drp1^Ser616^ in the tumor microenvironment had an increased risk on poor 5-year OS (HR = 3.821, 95% CI 1.459–10.008, *p* = 0.006, Table 2). These results indicated that phospho-Drp1^Ser616^ is a significant prognostic factor for survival outcome in LARC patients who treated with neoCRT. Moreover, after neoCRT treatment, we found that the high phospho-Drp1^Ser616^ was significantly observed in the metastatic lymph nodes (Fig. 6a), and associated with tumor relapse (Table S2).

**Table 2:** Univariate and multivariate analysis of 5-year OS in LARC

	5-Year OS					
	Univariate			Multivariate		
	HR	95% CI	*p* value	HR	95% CI	*p* value
*Pre-neoCRT (n* *=* *106)*						
pN stage (positive vs. negative)	2.611	1.131–6.026	0.025*	2.858	1.041–7.848	0.042*
Clinical response (poor response vs. good response)	2.161	0.907–5.153	0.082	1.006	0.324–3.126	0.991
TRG (poor response vs. good response)	2.115	0.913–4.895	0.08	2.135	0.817–5.581	0.122
Drp1 (high vs. low)	1.136	0.491–2.629	0.766	1.278	0.536–3.045	0.58
p-Drp1^Ser616^ (high vs. low)	3.164	1.238–8.089	0.016*	3.821	1.459–10.008	0.006*
*Pre-neoCRT (n* *=* *93)*						
pN stage (positive vs. negative)	2.605	1.079–6.291	0.033*	2.854	1.025–7.948	0.045*
Clinical response (poor response vs. good response)	2.206	0.848–5.743	0.105	1.075	0.333–3.470	0.904
TRG (poor response vs. good response)	2.068	0.861–4.969	0.104	2.230	0.830–5.992	0.112
Drp1 (high vs. low)	1.219	0.507–2.929	0.658	1.541	0.613–3.874	0.357
p-Drp1^Ser616^ (high vs. low)	3.065	1.177–7.982	0.022*	3.564	1.331–9.541	0.011*

pN stage: positive (Stage 1a + 1b + 2) vs. negative (Stage 0 + X); Clinical response: Good response (complete response and partial response) vs. poor response (stable disease and progression disease); TRG: Good response (TRG 3–4) vs. poor response (TRG 1–2); p-Drp1: high (grade 2 + 3) vs. low (grade 0 + 1)

Furthermore, we found a substantial proportion of RAGE-G82S polymorphism (36/106 [34%]) in patients with LARC. Patients with RAGE-G82S polymorphism did not correlated with high phospho-Drp1 ^Ser616^ before neoCRT treatment (*p* = 0.84, Table 3). But patients with RAGE-G82S polymorphism displayed high phospho-Drp1 ^Ser616^ after neoCRT treatment (*p* = 0.05, Table 3), suggesting that patients with RAGE-G82S polymorphisms may have increased Drp1 activation to promote chemoresistance and tumor regrowth after chemoradiotherapy treatment, leading to poor survival outcome in LARC. Taken together, these results demonstrated that HMGB1 released from dying cancer cells after neoCRT may enhance regrowth and metastasis of remnant cancer cells via RAGE-ERK-Drp1 activation.

**Table 3:** Correlation between p-Drp1 and RAGE SNP in pre-neoCRT biopsies and post-neoCRT surgical tissues of LARC

Variable	Cases	High-pDrp1^Ser616^		*p* value
		OR	95% CI	
*Pre-neoCRT treatment (n* *=* *106)*				
RAGE				0.84
WT	70	1		
Variant	36	1.121	0.5015–2.507	
*Post-neoCRT treatment (n* *=* *93)*				
RAGE				0.05*
WT	59	1		
Variant	34	2.478	1.025–5.989	

## Discussion

Most CRC patients develop resistance to chemotherapy drugs, such as oxaliplatin, which is a significant challenge in the treatment of colon cancer. This phenomenon has been attributed to different mechanisms, including dysfunctional membrane transport, resistance to apoptosis, and DNA repair mechanisms. In this study, we showed that Drp1-mediated mitochondria fission, which results in autophagy, is a significant contributor to drug resistance in CRC. Following chemotherapy treatment, the release of HMGB1 is induced, and HMGB1 can exert autocrine or paracrine effects that activate Drp1 and lead to chemoresistance and tumor recurrence (Fig. 6f). Moreover, the inhibition of mitochondria fission via Drp1, the blockage of the release of HMGB1 or the blockage of the HMGB1 receptor RAGE enhance the sensitivity of anti-cancer agents in CRC.

Cancer cells respond to chemotherapy in a variety of ways, ranging from the activation of survival pathways to the initiation of cell death. Consistent with previous studies that identified a cytoprotective role of autophagy, increased autophagy is observed following exposure to chemotherapy drugs^35,36^. Here, we reported that mitochondria fission by Drp1 hyperactivation is required for autophagy and chemoresistance. Because Drp1 has been shown to be involved in several cellular processes, such as mitochondrial energetics to sustain tumor development, stem cell maintenance, promotion of metastasis and cell proliferation, Drp1-driven mitochondrial fission has been considered a vital factor in the context of tumorigenesis. Several emerging studies have focused on the relationship between mitochondrial dynamics and cell fate determination^12,13,37,38^. Serasinghe et al. reported that ERK-mediated Drp1 S616 phosphorylation in mitochondrial division was necessary for RAS-induced transformation. The disruption of Drp1 activity was shown to promote mitochondrial fusion and inhibit tumor growth^13^. Moreover, recent studies have shown that mitochondrial fission via Drp1 significantly promoted cell survival by increasing autophagy, resulting in a resistance to apoptosis in HCC^10^ and chemoresistance in acute lymphoblastic leukemia (ALL)^11^. Cai et al. revealed that the remarkable elevation of the activating phosphorylation of Drp1 at S616 decreased the mitochondrial ROS levels, resulted in a pro-glycolytic metabolism shift and increased drug resistance^11^. Collectively, these results and our present findings indicate that Drp1-mediated mitochondrial dynamics are associated with increasing autophagy, resulting in resistance to cell death and chemoresistance.

HMGB1 has been reported as a DAMP for immunogenic cell death (ICD) during chemotherapy and radiotherapy, and its release from necrotic cancer cells may activate the innate immune system to promote host anticancer immunity. However, extracellular HMGB1 is known to act as a growth factor that activates mitogen-activated protein kinase (MAPK) and thereby enhances the regrowth, metastasis, and chemoresistance of remnant cancer cells that survived prior chemotherapy via RAGE^15,19,39–43^. Thus, the role of chemotherapy-associated HMGB1 is thought to depend on the balance of these different effects of HMGB1. Indeed, the knockdown of RAGE or HMGB1 increased cell death and restored the chemosensitivity of the cancer cells in vivo and in vitro^21^, indicating that this this knockdown may lead to the induction of cancer progression and drug resistance. The ability of released HMGB1 to promote chemoresistance in cancer cells, such as neuroblastoma^44^, lung adenocarcinoma^18^, prostate cancer^45^, CRC^17^, and leukemia^46^, is reportedly due to autophagy^47^. Consistently, we found that OXP, CAP, and CPT-11 promote the enhanced expression and secretion of HMGB1 in human colorectal cancer cells. HMGB1 has been reported to be a critical regulator of autophagy. Cytosolic HMGB1 is an inducer of starvation-mediated autophagy by binding to Beclin 1 in colorectal cancer^48^. In addition, extracellular HMGB1 induces autophagy via its receptor RAGE in colorectal and pancreatic cancer cells^21,49^. HMGB1-mediated autophagy modulates the sensitivity of colorectal cancer cells to Oxaliplatin via the MEK/ERK signaling pathway^17^. This hypothesis is supported by our findings in which HMGB1 was associated with autophagy and chemoresistance via RAGE/MEK/ERK/Drp1 in colorectal cancer. Moreover, we found that the expression of Drp1 and p-Drp1 was positively correlated with patients carrying germ-line single-nucleotide polymorphisms (SNPs) of RAGE with G82S (rs2070600) after neoCRT treatment. RAGE-G82S has been reported to exhibit potentially enhanced ligand-binding affinity, such as to HMGB1^24,50^, which supports our finding in which HMGB1 triggered Drp1 phosphorylation *via* RAGE.

Our results suggested that patients with RAGE-G82S polymorphisms may have high affinity for the ligand, such as HMGB1, which activates ERK1/2 to phosphorylate Drp1 for mitochondria fission and autophagy. Eventually, Drp1-mediated mitochondria fission promotes chemoresistance and tumor regrowth, leading to poor survival outcome in LARC.

The data reported here indicate the potential of extracellular HMGB1 released from colorectal cancer cells to exert a paracrine effect on surviving cancer cells enabling them to resist chemoradiotherapy. Targeting the receptor RAGE and the downstream effector Drp1 in cancer cells may prevent or inhibit the development of drug resistance. In contrast, there is evidence that chemotherapeutic drug-induced HMGB1 can mediate the activation of innate immunity and tumor clearance. Thus, caution must be exercised because of the potential positive and negative aspects of HMGB1 expression during different phases of tumor development and treatment.

These results showed that the potential of extracellular HMGB1 released from colorectal cancer cells to exert a paracrine effect on surviving cancer cells enabling them to resist chemoradiotherapy via RAGE-ERK-Drp1 signaling pathway.

## Electronic supplementary material

Supplementary information
