# Supplementary material for: HMGB1 promotes ERK-mediated mitochondrial Drp1 phosphorylation for chemoresistance through RAGE in colorectal cancer
Source: Cell Death Dis. 2018 Sep 26;9(10):1004. doi: 10.1038/s41419-018-1019-6 (PMC6158296; doi:10.1038/s41419-018-1019-6)
Supplement: Supplementary file 1 — Supplementary information [file 41419_2018_1019_MOESM1_ESM.doc]

**Supplementary information**


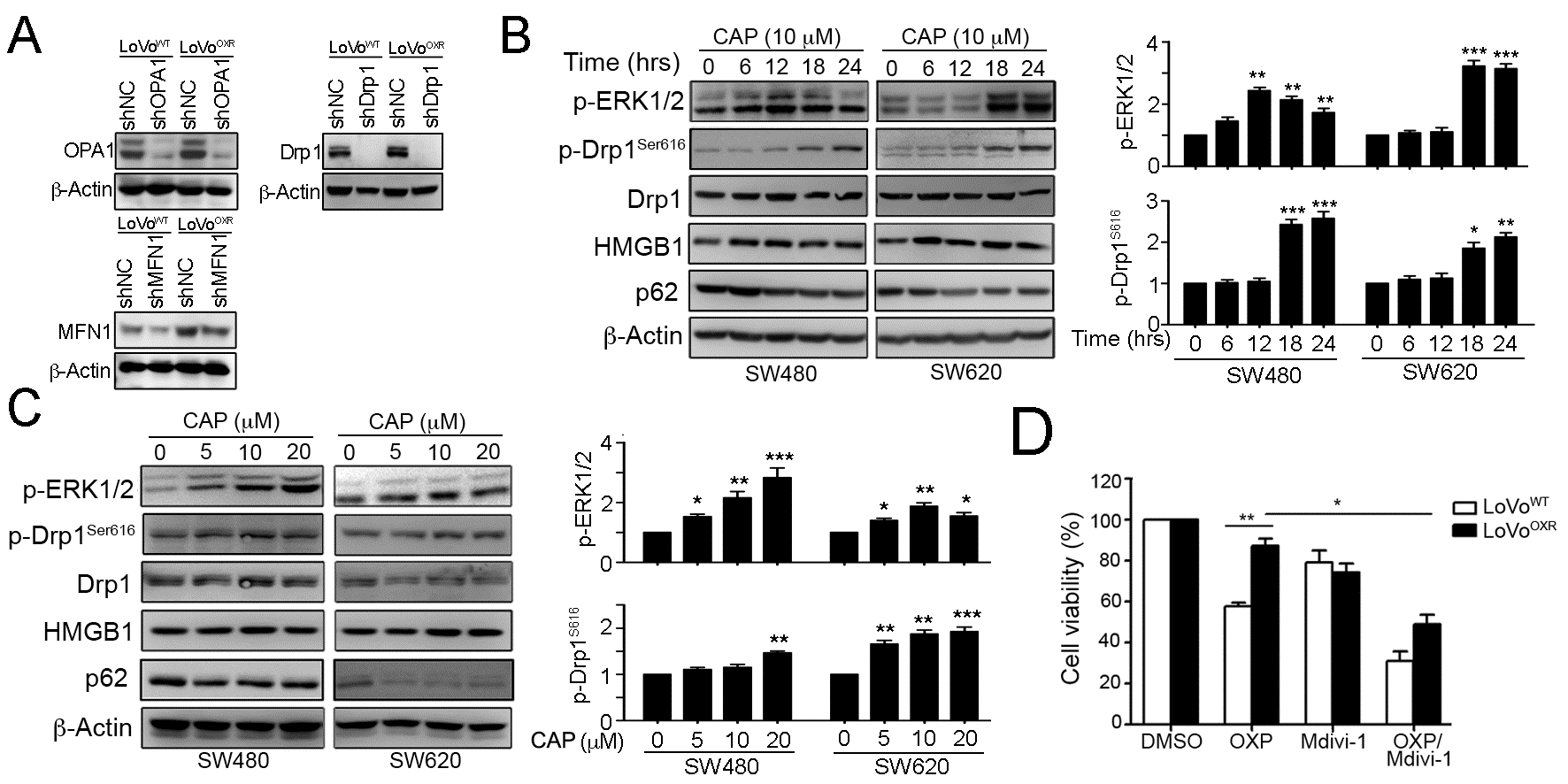


**Supplementary figure 1. Drp1 becomes hyperactivated following cytotoxic insults and promotes autophagy.**

(A) The knockdown efficiency of lentivirus carrying shRNA against OPA1, Drp1 and MFN1 in LoVo^WT^ and LoVo^OXR^ cell lines.

(B) SW480 and SW620 were treated with capecitabine (CAP, 10 μM) for diverse time. Cell lysate were analyzed by immunoblotting. Quantification of these results is shown (n=3). **p*<0.05, ***p*<0.01 and ****p*<0.001.

(C) SW480 and SW620 were treated with various concentration of capecitabine (CAP) for 24 hrs. Cell lysate were analyzed by immunoblotting. Quantification of these results is shown (n=3). **p*<0.05, ***p*<0.01 and ****p*<0.001.

(D) Cells were treated with OXP (15 μM), Mdivi-1 (50 μM) and OXP/Mdivi-1 for 48 hrs. The cell viability was analyzed by MTT assay. **p*<0.05 and ***p*<0.01.

These data were obtained from three independent experiments, and the values represent the means ±S.D.

| **Table S1. Patient and tumor characteristics in pre-neoCRT biopsy of LARC patients (N=106)** | | | | | | | |
| --- | --- | --- | --- | --- | --- | --- | --- |
| Clinicopathological characteristics | Total cases | Drp1 | | *p* value | p-Drp1^Ser616^ | | *p* value |
|  |  | High | Low |  | High | Low |  |
|  | 106 | 45 | 61 |  | 51 | 55 |  |
| Age |  |  |  | 0.5341 |  |  | 0.5369 |
| <65 | 72 | 29 | 43 |  | 33 | 39 |  |
| >65 | 34 | 16 | 18 |  | 18 | 16 |  |
| Sex |  |  |  | 0.8321 |  |  | 0.1504 |
| Male | 74 | 32 | 41 |  | 39 | 35 |  |
| Female | 32 | 13 | 19 |  | 12 | 20 |  |
| pN |  |  |  | 0.5285 |  |  | 0.5544 |
| Negative | 74 | 33 | 41 |  | 37 | 37 |  |
| Positive | 32 | 12 | 20 |  | 14 | 18 |  |
| Pathologic TNM stage |  |  |  | 0.5285 |  |  | 0.5544 |
| 0-II | 74 | 33 | 41 |  | 37 | 37 |  |
| III-IV | 32 | 12 | 20 |  | 14 | 18 |  |
| Chemotherapy |  |  |  | 0.656 |  |  | 0.709 |
| Xeloda | 58 | 26 | 32 |  | 28 | 30 |  |
| UFT | 41 | 16 | 25 |  | 20 | 21 |  |
| FOLFOX | 1 | 0 | 1 |  | 1 | 0 |  |
| 5-Fu | 5 | 3 | 2 |  | 2 | 3 |  |
| Xeloda+ UFT | 1 | 0 | 1 |  | 0 | 1 |  |
| TRG |  |  |  | 0.4214 |  |  | 0.3009 |
| 4 | 13 | 3 | 10 |  | 8 | 5 |  |
| 3 | 61 | 29 | 32 |  | 28 | 33 |  |
| 2 | 21 | 9 | 12 |  | 12 | 9 |  |
| 1 | 11 | 4 | 7 |  | 3 | 8 |  |
| Clinical response |  |  |  | 0.1379 |  |  | 0.4754 |
| CR | 12 | 3 | 9 |  | 7 | 5 |  |
| PR | 44 | 23 | 21 |  | 19 | 25 |  |
| SD | 43 | 18 | 25 |  | 23 | 20 |  |
| PD | 7 | 1 | 6 |  | 2 | 5 |  |
| Tumor relapse |  |  |  | 0.8084 |  |  | **0.0073*** |
| No | 79 | 33 | 46 |  | 32 | 47 |  |
| Yes | 27 | 12 | 15 |  | 19 | 8 |  |
| pN stage: negative (Stage 0+X) vs positive (Stage 1a+1b+2); Clinical response: Good response (complete response and partial response) vs Poor response (stable disease and progression disease) | | | | | | | |

| **Table S2. Clinicopathologic parameters with tumor relapse in post-neoCRT surgical tissues of LARC (N=93)** | | | | |
| --- | --- | --- | --- | --- |
| **Variable** | **Total cases** | **p-Drp1^Ser616^** | | ***p* value** |
|  |  | **High** | **Low** |  |
|  | 93 | 50 | 43 |  |
| Tumor relapse |  |  |  | **0.0002***** |
| Yes | 23 | 20 | 3 |  |
| No | 70 | 30 | 40 |  |
